# Impact of Seizures and Status Epilepticus on Outcome in Patients with Aneurysmal Subarachnoid Hemorrhage

**DOI:** 10.1007/s12028-022-01489-0

**Published:** 2022-04-12

**Authors:** Stefan Yu Bögli, Sophie Wang, Natalia Romaguera, Valerie Schütz, Omar Rafi, Marco Gilone, Emanuela Keller, Lukas L. Imbach, Giovanna Brandi

**Affiliations:** 1 Institute for Intensive Care Medicine, University Hospital Zurich, Frauenklinikstrasse 26, 8091 Zurich, Switzerland; 2 Department of Neurology, University Hospital Zurich, Zurich, Switzerland; 3 Clinical Neuroscience Center, University Hospital and University of Zurich, Zurich, Switzerland; 4 Department of Neurosurgery and Neurotechnology, Eberhard Karls University Tübingen, Tübingen, Germany; 5 Department of Neurosurgery, University Hospital Zurich, Zurich, Switzerland; 6 Swiss Epilepsy Center, Klinik Lengg AG, Zurich, Switzerland

**Keywords:** Status epilepticus, Aneurysmal subarachnoid hemorrhage, Outcome

## Abstract

**Background:**

We aimed to evaluate the association between seizures as divided by timing and type (seizures or status epilepticus) and outcome in patients with aneurysmal subarachnoid hemorrhage (aSAH).

**Methods:**

All consecutive patients with aSAH admitted to the neurocritical care unit of the University Hospital Zurich between 2016 and 2020 were included. Seizure type and frequency were extracted from electronic patient files.

**Results:**

Out of 245 patients, 76 experienced acute symptomatic seizures, with 39 experiencing seizures at onset, 18 experiencing acute seizures, and 19 experiencing acute nonconvulsive status epilepticus (NCSE). Multivariate analysis revealed that acute symptomatic NCSE was an independent predictor of unfavorable outcome (odds ratio 14.20, 95% confidence interval 1.74–116.17, *p* = 0.013) after correction for age, Hunt-Hess grade, Fisher grade, and delayed cerebral ischemia. Subgroup analysis showed a significant association of all seizures/NCSE with higher Fisher grade (*p* < 0.001 for acute symptomatic seizures/NCSE, *p* = 0.031 for remote symptomatic seizures). However, although acute seizures/NCSE (*p* = 0.750 and 0.060 for acute seizures/NCSE respectively) were not associated with unfavorable outcome in patients with a high Hunt-Hess grade, they were significantly associated with unfavorable outcome in patients with a low Hunt-Hess grade (*p* = 0.019 and *p* < 0.001 for acute seizures/NCSE, respectively).

**Conclusions:**

Acute symptomatic NCSE independently predicts unfavorable outcome after aSAH. Seizures and NCSE are associated with unfavorable outcome, particularly in patients with a low Hunt-Hess grade. We propose that NCSE and the ictal or postictal reduction of Glasgow Coma Scale may hamper close clinical evaluation for signs of delayed cerebral ischemia, and thus possibly leading to delayed diagnosis and therapy thereof in patients with a low Hunt-Hess grade.

## Introduction

Seizures and nonconvulsive status epilepticus (NCSE) are a common complication in patients with aneurysmal subarachnoid hemorrhage (aSAH). They are commonly divided into onset (occurring within 24 h after hemorrhage), acute (occurring within the first 7 days after hemorrhage, also termed “provoked seizures/NCSE”), and remote symptomatic (occurring after the initial acute stage of disease, leading to the diagnosis of epilepsy) [1, 2]. Although onset seizures occur in around 4–19% [3–5] of patients with aSAH, early seizures occur in around 1–11.7% [6–9]. Both onset and early seizures were early on implied to predict unfavorable outcome because of their correlation to rebleeding [3, 10] before early surgical therapy was routinely available. This result could not consistently be replicated in later studies [3, 5, 9, 11, 12]. Recently, however, seizure burden (as a quantification of the duration of subclinical seizures) has been found to impair cognitive outcome after 3 months, implying that possibly only higher functions are impaired by recurrent seizures [8]. Late seizures (and thus diagnosis of epilepsy) occur in around 1–30% of patients [9, 13, 14] (depending on the length of follow-up). NCSE occurs in 3–15% [15, 16] of patients with aSAH. Unlike self-limiting seizures, its presence has generally been found to be detrimental, with unfavorable outcome in up to 92% of patients [17, 18]. According to the suggested definition of the International League Against Epilepsy (ILAE), seizures and NCSE have a period of 7 days during which their occurrence is deemed provoked in a multitude of diseases (e.g., stroke, traumatic brain injury, infectious/autoimmune central nervous system disease, etc.) [1]. In aSAH, this definition is hampered by the occurrence of delayed cerebral ischemia (DCI) up to 14 days after ictus. Thus, studies have commonly averted from reporting acute symptomatic and remote symptomatic seizures but differentiated early (ranging from inclusion of onset seizures, up to 24–48 h after ictus, up to 1 or 2 weeks after ictus, or even up to hospital discharge) and late seizures (7–14 days after ictus or after discharge) [6, 19]. This variability in definition might considerably impair and alter outcome prediction. Furthermore, studies commonly combine self-limiting seizures and NCSE within the reports. The primary aim of this study was to provide an in-depth description of seizures and NCSE in a current cohort of patients with aSAH by rigorous retrospective analysis with respect to their timing and type. A secondary aim was to provide a quantification of their predictive value for outcome prediction in aSAH.

## Materials and Methods

The study was approved by the local ethics committee (Cantonal Ethics Commission Zurich, Kantonale Ethikkomission 2019-00713) and was in accordance with the ethical standards laid down in the 2013 Declaration of Helsinki for research involving human participants. Informed consent was received before inclusion by the patient or their legal medical representative.

The patients were selected from a prospective database of consecutive patients who were admitted between 2016 and 2020 to the neurocritical care unit of the University Hospital Zurich because of an aSAH. Only patients with imaging evidence of a ruptured intracranial aneurysm were included. Patients with immediate withdrawal of life-sustaining therapy because of the severity of the disease and patients with a preexisting diagnosis of epilepsy at onset were excluded. Data collection was performed by scanning the electronic health records for demographic characteristics, clinical and radiological information (Fisher grade, location of ruptured aneurysm, presence of other unruptured aneurysms, presence of intracerebral as well as intraventricular or subdural hemorrhage including their location, and presence of hydrocephalus), treatment modality (clipping, coiling or flow diverter–based therapy), clinical course (occurrence of ventriculostomy-related infection, vasospasm, or DCI), and outcome data. The radiological findings aside from DCI and vasospasm were extracted from the first computed tomography (CT) (including CT angiography) imaging. DCI was defined as a cerebral infarction on CT scans or magnetic resonance images, excluding infarction caused by other causes (such as endovascular treatment or clipping) and infarction already present within the CT scan 24–48 h after aneurysm securing [20]. The clinical correlate of DCI (occurrence of focal neurological impairment or Glasgow Coma Scale [GCS] decrease of 2 or more points for at least 1 h) was also excluded to reduce the bias caused by the retrospective design of this study. Transcranial Doppler ultrasound monitoring was performed daily. The cerebral flow velocities were not used for the diagnosis of vasospasm but only triggered the performance of a CT scan, including angiography (in case of increasing or increased flow velocities). The diagnosis of vasospasm was reserved for cases with respective description on CT, magnetic resonance, or digital subtraction angiography. All patients received nimodipine as a vasospasm prophylaxis. The dosage was either 60 mg per 4 h orally or 2 mg per hour intravenously in cases with relevant hemodynamic instability (i.e., to be able to quickly reduce the dosage). The Charlson Comorbidity Index was assessed to evaluate relevant comorbidity. The Charlson Comorbidity Index correlates with mortality and consists of differently weighted medical conditions [21]. Outcome is presented by using the Glasgow Outcome Scale Extended (GOSE) extracted from routine follow-up consultations at 3 months (which include a neurological examination and a description of current occupation, including the percentage of working capability).

Seizures (that are regularly assessed and noted during the hospital stay and during each follow-up consultation at 3 months and at 12 months), including their type and timing, were extracted from the medical reports. Acute symptomatic seizures/NCSE were defined to have occurred within 7 days after hemorrhage. Remote symptomatic seizures/NCSE were defined to have occurred 8 days or later after hemorrhage (in the absence of other seizure provoking factors). Provoked seizures due to complication were defined as seizures that occurred because of clear seizure provoking factors (e.g., severe metabolic or electrolyte disturbance, acute progressive hydrocephalus, rebleed, etc.). Electroencephalogram (EEG) reports were extracted and verified by two experienced neurologists (LI and SB) through reevaluation of the EEGs. NCSE was diagnosed by using the Salzburg criteria [22]. Epilepsy was diagnosed by the occurrence of remote symptomatic seizures/NCSE 8 days or later after initial hemorrhage. Furthermore we provide the number of patients who had their first remote symptomatic seizure/NCSE between 8 and 14 days after hemorrhage (the vasospasm phase). At the neurocritical care unit of the University Hospital Zurich, EEGs are performed on the basis of the judgment of the treating physician alone. EEGs last for at least 20 min. Anesthetics are stopped before the start of EEG acquisition (i.e., midazolam is stopped at least 6 h before acquisition, propofol at least 20 min before acquisition, no inhalative anesthetics are used). In case signs of sedation were found during EEG inspection, the EEG is repeated later on. Continuous EEG monitoring is only used in cases with refractory NCSE or need for deep sedation because of increased intracerebral pressure. Prophylactic antiseizure medication (ASM) is not routinely administered at our institution. In case of seizures (either with clinical correlate or subclinical seizure found during EEG monitoring) or diagnosis of NCSE, ASM was started or increased (in case of recurrent seizures or persistent NCSE). Epileptiform discharges alone did not lead to an escalation of ASM.

### Statistical Analysis

Statistical analysis was performed using SPSS version 25. Descriptions are reported as counts/percentages, means ± standard deviations, or as medians including the interquartile ranges, as appropriate. For the analysis of predisposing factors for an unfavorable outcome, patient characteristics were dichotomized depending on the GOSE, with 5–8 being favorable and 1–4 being unfavorable outcomes. All continuous data were tested for normality by using Shapiro–Wilk’s test. Categorical variables were compared with Pearson’s *χ*^2^ or Fisher’s exact test, continuous/ordinal variables using Student’s *t*-test or Mann–Whitney *U*-test for parametric and nonparametric data, respectively, when appropriate. Multivariate logistic regression was performed to ascertain the effects of found risk factors on the likelihood of unfavorable outcome. Bonferroni correction was applied to correct for multiple comparisons. As a measure of the overall discriminatory ability of the models, the area under the receiver operating characteristic (ROC) curve, including 95% confidence interval (CI), is reported. Lastly, subgroup analyses for differences between patients with low/high Fisher grade (1–2 vs. 3–4) and low/high Hunt-Hess grade (1–2 vs. 3–5) in respect to their outcome was performed.

## Results

### Patient Characteristics

**Fig. 1:**
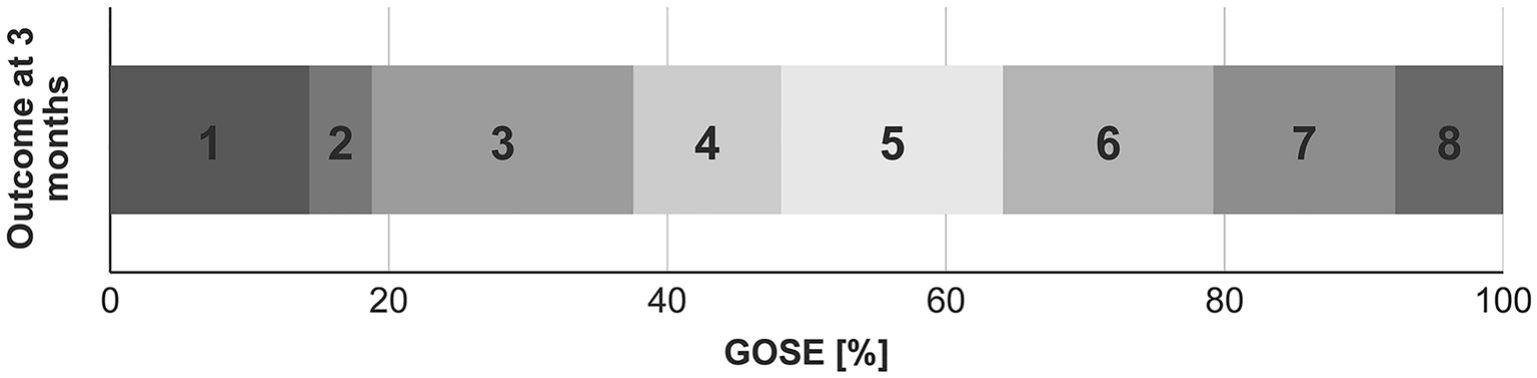
Three-month outcome as evaluated by using the Glasgow Outcome Scale Extended (GOSE)

A total of 245 patients with aSAH were identified between 2016 and 2020. The outcome (3-month GOSE) is shown in Fig. 1. Twenty-seven patients died during the hospital stay. Among these, life-sustaining therapy was withdrawn in 18 patients. Patient characteristics stratified by favorable or unfavorable outcome can be found in Table 1.

**Table 1:** Patient characteristics dichotomized by outcome

Parameter	All patients	Outcome		*p* value
		Favorable, *n* = 127 (51.8%)	Unfavorable, *n* = 118 (48.1%)	
Age (y)	57 ± 12.8	54 ± 11.6	60 ± 13.2	** < 0.001**
Female sex	155 (63.3)	72 (56.7)	83 (70.3)	**0.027**
Charlson Comorbidities Index	0 (0–2)	0 (0–1)	0 (0–2)	0.085
length of stay at the ICU (d)	20 ± 26.6	18 ± 33.4	23 ± 16.1	0.080
Hunt-Hess grade				** < 0.001**
1	60 (24.7)	48 (38.1)	12 (10.3)	
2	57 (23.5)	36 (28.6)	21 (17.9)	
3	45 (18.5)	24 (19.0)	21 (17.9)	
4	41 (16.9)	12 (9.5)	29 (24.8)	
5	40 (16.5)	6 (4.8)	34 (29.1)	
Location of ruptured aneurysm				
Anterior circulation	207 (84.5)	108 (85.0)	99 (83.9)	0.805
Posterior circulation	38 (15.5)	19 (15.0)	19 (16.1)	
Other unruptured aneurysms	72 (29.4)	30 (23.6)	42 (35.6)	**0.040**
Fisher grade				** < 0.001**
1	10 (4.1)	10 (8.0)	0 (0.0)	
2	18 (7.4)	16 (12.8)	2 (1.7)	
3	113 (46.5)	71 (56.8)	42 (35.6)	
4	102 (42.0)	28 (22.4)	74 (62.7)	
Intracerebral hemorrhage (ICH)	80 (32.7)	23 (18.1)	57 (48.3)	** < 0.001**
Multilobar ICH	19 (7.8)	5 (3.9)	14 (11.9)	**0.020**
Location of ICH				
Frontal lobe	51 (20.8)	13 (10.2)	38 (32.2)	** < 0.001**
Temporal lobe	35 (14.3)	11 (8.7)	24 (20.3)	**0.009**
Parietal lobe	12 (4.9)	2 (1.6)	10 (8.5)	**0.012**
Occipital lobe	2 (0.8)	0 (0.0)	2 (1.7)	0.231
Other (basal ganglia, brainstem)	8 (3.3)	4 (3.1)	4 (3.4)	1.000
Intraventricular hemorrhage	163 (66.8)	65 (51.6)	98 (83.1)	** < 0.001**
Blood in the basal cisterns	163 (66.8)	79 (62.7)	84 (71.2)	0.159
Subdural hematoma	25 (10.2)	6 (4.7)	19 (16.1)	**0.003**
Hydrocephalus	118 (48.2)	43 (33.9)	75 (63.6)	** < 0.001**
Clinical course				
Treatment modality				
Clipping	115 (46.9)	62 (48.8)	53 (44.9)	0.629
Coiling	123 (50.2)	60 (47.2)	63 (53.4)	0.405
Flow-diverter	7 (2.9)	5 (3.9)	2 (1.7)	0.449
Ventriculostomy-related infection	32 (13.1)	13 (10.2)	19 (16.1)	0.173
Vasospasm	154 (62.9)	74 (58.3)	80 (67.8)	0.123
Delayed cerebral ischemia	61 (24.9)	23 (18.1)	38 (32.2)	**0.010**

Bold values indicate statistical signifcance at *p* < 0.05

Patient characteristics for all patients or dichotomized by outcome. Values are indicated as number (percentage), mean ± standard deviation, or median (interquartile range). *p* values are based on univariate analysis

ICH, intracerebral haemorrhage; ICU, intensive care unit

Significant differences in age, sex, Hunt-Hess grade, Fisher grade, presence of intracerebral hemorrhage (especially if entailing the frontal lobe or being multilobar), intraventricular hemorrhage, subdural hemorrhage, hydrocephalus, and DCI depending on outcome were found.

**Fig. 2:**
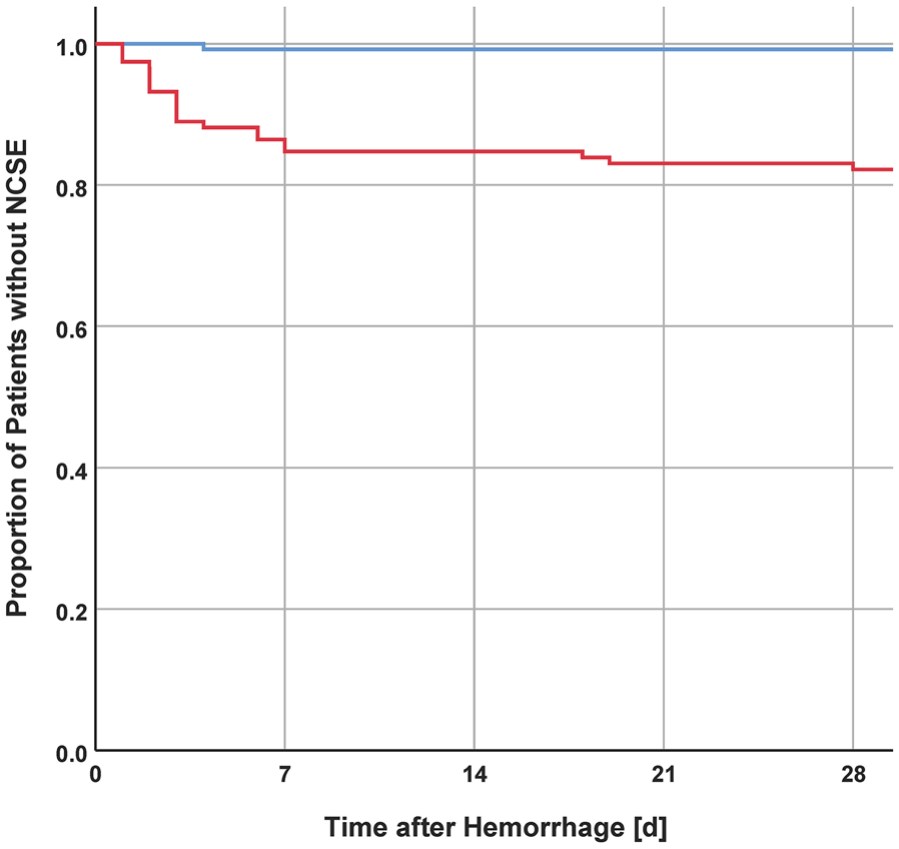
Kaplan–Meier curve: time to occurrence of first nonconvulsive status epilepticus (NCSE). The proportion of patients without NCSE during the first 4 weeks after hemorrhage is shown dichotomized by outcome (blue = favorable, red = unfavorable) (Color figure online)

Incidence of seizures/NCSE are summarized in Table 2. A total of 76 (31.0%) patients experienced acute symptomatic seizures, with 39 (15.9%) being onset seizures, 18 (7.3%) being acute symptomatic self-limiting seizures, and 19 (7.8%) being acute NCSE. Significant differences in outcome were found for patients with acute self-limiting seizures (*p* = 0.034) and acute NCSE (*p* < 0.001). Epilepsy was diagnosed in 40 (16.3%) patients. Six patients had their first remote symptomatic seizure between 8 and 14 days after onset of aSAH. Figure 2 shows the Kaplan–Meier curve of the time to occurrence of first NCSE (irrespective of acute or remote origin) dichotomized by outcome. The percentage of patients receiving an EEG (52.9%, 55.0%, 57.6%, 70.4%, and 66.7% for the years between 2016 and 2020, respectively) and the number of NCSE diagnosed (8.8%, 7.5%, 6.7%, 11.1%, and 11.1% for the years between 2016 and 2020, respectively) increased over the years.

**Table 2:** Seizures/epilepsy dichotomized by outcome

Parameter	All patients	Outcome at 3 months		*p* value
		Favorable, *n* = 127 (51.8%)	Unfavorable, *n* = 118 (48.1%)	
Seizure at onset	39 (15.9)	15 (11.8)	24 (20.3)	0.068
Acute symptomatic seizure	18 (7.3)	5 (3.9)	13 (11.0)	**0.034**
Acute symptomatic NCSE	19 (7.8)	1 (0.8)	18 (15.3)	** < 0.001**
ASM at discharge	70 (32.1)	23 (18.1)	47 (39.8)	** < 0.001**
EEG during first 7 days	146 (59.6)	48 (37.8)	98 (83.1)	** < 0.001**
Remote symptomatic seizure	22 (9.0)	6 (4.7)	16 (13.6)	**0.016**
Remote symptomatic NCSE	21 (8.6)	1 (0.8)	20 (16.9)	** < 0.001**
Epilepsy	40 (16.3)	7 (5.5)	33 (28.0)	** < 0.001**
First remote symptomatic seizure 8–14 days after aSAH	6 (2.4)	0 (0.0)	6 (2.4)	** < 0.012**
ASM after 1 year	49 (25.5)	13 (10.2)	36 (30.5)	** < 0.001**
Provoked seizure due to complication	7 (2.9)	1 (0.8)	6 (5.1)	0.058

Bold values indicate statistical signifcance at p < 0.05

Seizure characteristics for all patients or dichotomized by outcome. Values are indicated as number (percentage). *P*-values based on univariate analysis

aSAH, aneurysmal subarachnoid haemorrhage; ASM, antiseizure medication; EEG, electroencephalography; NCSE, nonconvulsive status epilepticus

### Outcome Modeling

A multivariate logistic regression was performed to ascertain the effects of age, Hunt-Hess grade, Fisher grade, presence of subdural hematoma, hydrocephalus as well as DCI on the prediction of unfavorable outcome. Although age, Hunt-Hess grade, Fisher grade, and DCI retained their significance, the other factors did not and are left out in the following models (Table 3). The base model without these factors stayed statistically significant (*p* < 0.001). The area under the ROC curve was 0.84 (CI 0.79–0.89).

**Table 3:** Multivariate logistic regression modeling for the prediction of unfavorable outcome

Parameter	Model 0	Model 1	Model 2
Age	1.06 (1.03–1.09)*	1.05 (1.02–1.08)*	1.04 (1.02–1.07)*
Hunt-Hess grade	1.84 (1.42–2.38)*	1.86 (1.43–2.42)*	1.79 (1.38–2.32)*
Fisher grade	2.85 (1.64–4.96)*	2.74 (1.55–4.86)*	3.00 (1.70–5.28)*
Delayed cerebral ischemia	2.98 (1.41–6.28)*	3.12 (1.47–6.64)*	2.74 (1.28–5.86)*
Acute symptomatic NCSE	–	14.20 (1.74–116.17)*	–
Remote symptomatic NCSE	–	–	20.60 (1.80–235.41)*
ROC (95% CI)	0.84 (0.79–0.89)	0.86 (0.81–0.90)	0.86 (0.81–0.90)

Multivariate logistic regression models for the prediction of unfavorable outcome prediction (dependent variable) depending on age, Hunt-Hess grade, Fisher grade, delayed cerebral ischemia, and acute symptomatic NCSE or remote symptomatic NCSE. Values are indicated as odds ratio (95% confidence interval)

CI, confidence interval; NCSE, nonconvulsive status epilepticus; ROC, receiver operating characteristic

^*^Significant values (*p* < 0.05)

Acute symptomatic NCSE, as well as remote symptomatic NCSE significantly predicted unfavorable outcome in univariate binomial logistic regression with odds ratios (OR) of 22.68 (CI 2.98–172.81, *p* = 0.003) and 25.71 (CI 3.39–194.94, *p* = 0.002), respectively. Outcome prediction based on acute symptomatic NCSE alone provided a low sensitivity of 15.3% and a high specificity of 99.2%, leading to a positive predictive value for prediction of unfavorable outcome of 94.7% and a negative predictive value of 55.8%. Similarly, outcome prediction based on remote symptomatic NCSE alone provided a low sensitivity of 16.9% and a high specificity of 99.2%, leading to a positive predictive value of 95.2% and a negative predictive value of 56.3%.

Acute symptomatic NCSE and remote symptomatic NCSE were added separately to the base model. Both factors retained their significance in the model with OR of 14.20 (CI 1.74–116.17, *p* = 0.013) and 20.60 (CI 1.80–235.41, *p* = 0.015), respectively. The ROC with inclusion of these factors increased to 0.86 (CI 0.81–0.90) and 0.86 (CI 0.81–0.90) for acute symptomatic as well as remote symptomatic NCSE respectively. Acute symptomatic (*p* = 0.109) and remote symptomatic seizures (*p* = 0.088) did not retain their predictive significance if added to the base model.

### Subgroup Analysis

To evaluate differences in occurrence and characteristics of seizures/NCSE by initial clinical presentation as well as initial imaging, outcome dichotomized by Fisher grade or Hunt-Hess grade was performed (Tables 4, 5). As expected, seizures/NCSE primarily occurred in patients with a high Fisher grade. However, although seizures/NCSE were not significantly associated with unfavorable outcome in patients with a high Hunt-Hess grade, they were significantly associated with an unfavorable outcome in patients with a low Hunt-Hess grade.

**Table 4:** Subgroup analysis depending on Fisher grade and outcome

Parameter	Fisher grade 1–2			Fisher grade 3–4		
	Outcome			Outcome		
	Favorable	Unfavorable	*χ*^2^	Favorable	Unfavorable	*χ*^2^
	*n* = 26	*n* = 2	*p* value	*n* = 99	*n* = 116	*p* value
Seizure at onset	1 (3.8)	0 (0.0)	–	14 (14.1)	24 (20.7)	0.210
Acute symptomatic seizure	0 (0.0)	0 (0.0)	–	5 (5.1)	13 (11.2)	0.104
Acute symptomatic NCSE	0 (0.0)	0 (0.0)	–	1 (1.0)	18 (15.5)	** < 0.001**
ASM at discharge	2 (7.7)	0 (0.0)	–	21 (21.2)	45 (38.8)	** < 0.001**
EEG during first 7 days	3 (11.5)	0 (0.0)	–	44 (44.4)	96 (82.8)	** < 0.001**
Remote symptomatic seizure	1 (3.8)	0 (0.0)	–	5 (5.1)	16 (13.8)	**0.031**
Remote symptomatic NCSE	1 (3.8)	0 (0.0)	–	0 (0.0)	20 (17.2)	** < 0.001**
Epilepsy	2 (7.7)	0 (0.0)	–	5 (5.1)	33 (28.4)	** < 0.001**
First remote symptomatic seizure 8–14 days after aSAH	0 (0.0)	0 (0.0)	–	0 (0.0)	6 (5.2)	**0.032**
ASM after 1 year	1 (3.8)	1 (50.0)	–	12 (12.1)	35 (30.2)	** < 0.001**

Bold values indicate statistical signifcance at *p* < 0.05

Seizure characteristics dichotomized firstly by the Fisher grade and secondly by outcome. Favorable as well as unfavorable outcome are compared for each Fisher grade group separately. Comparison of the low Fisher grade group was left out due to the small number of patients with unfavorable outcome. Values are indicated as number (percentage). *p* values are based on univariate analysis

aSAH, aneurysmal subarachnoid haemorrhage; ASM, antiseizure medication; EEG, electroencephalography; NCSE, nonconvulsive status epilepticus

**Table 5:** Subgroup analysis depending on Hunt-Hess grade and outcome

Parameter	Hunt-Hess grade 1–2			Hunt-Hess grade 3–5		
	Outcome			Outcome		
	Favorable	Unfavorable	*χ*^2^	Favorable	Unfavorable	*χ*^2^
	*n* = 84	*n* = 33	*p* value	*n* = 42	*n* = 84	*p* value
Seizure at onset	6 (7.1)	5 (15.2)	0.288	9 (21.4)	19 (22.6)	0.880
Acute symptomatic seizure	2 (2.4)	5 (15.2)	**0.019**	3 (7.1)	8 (9.5)	0.750
Acute symptomatic NCSE	0 (0.0)	7 (21.2)	** < 0.001**	1 (2.4)	11 (13.1)	0.060
ASM at discharge	10 (11.9)	15 (45.5)	** < 0.001**	13 (31.0)	32 (38.1)	0.052
EEG during first 7 days	24 (28.6)	27 (81.8)	** < 0.001**	24 (57.1)	70 (83.3)	**0.001**
Remote symptomatic seizure	1 (1.2)	8 (24.2)	** < 0.001**	5 (11.9)	8 (9.5)	0.759
Remote symptomatic NCSE	1 (1.2)	5 (15.2)	**0.007**	0 (0.0)	15 (17.9)	**0.004**
Epilepsy	2 (2.4)	13 (39.4)	** < 0.001**	5 (11.9)	20 (23.8)	0.114
First remote symptomatic seizure 8–14 days after aSAH	0 (0.0)	2 (6.1)	0.078	0 (0.0)	4 (4.8)	0.300
ASM after 1 year	5 (6.0)	8 (24.2)	**0.001**	8 (19.0)	28 (33.3)	**0.001**

Bold values indicate statistical signifcance at *p* < 0.05

Seizure characteristics dichotomized first by Hunt-Hess grade and second by outcome. Favorable as well as unfavorable outcome are compared for each Hunt-Hess grade group separately. Values are indicated as number (percentage). *p* values are based on univariate analysis

aSAH, aneurysmal subarachnoid haemorrhage; ASM, antiseizure medication; EEG, electroencephalography; NCSE, nonconvulsive status epilepticus

## Discussion

This study provides a detailed description of the association of seizures/NCSE (divided into onset, acute symptomatic, and remote symptomatic) and outcome in a current cohort of patients with aSAH.

Early studies found seizures at onset to be associated with unfavorable outcome [3, 10]. Later studies as well as our current study could not replicate this result probably due to reduced rates of rebleeding [3, 5, 9, 11, 12]. Furthermore, acute self-limiting seizures were not significantly associated with unfavorable outcome in our patient group. Conversely, acute symptomatic NCSE was associated with unfavorable outcome even after correction for the known risk factors (including Hunt-Hess grade, Fisher grade, and DCI). Acute symptomatic NCSE occurred in 1 versus 18 patients with favorable/unfavorable outcome, respectively, leading to a high positive predictive value for unfavorable outcome. Conversely, because of the low number of patients affected by NCSE (8%) negative predictive value remained low (altogether leading to a relatively large CI of NCSE OR in the multivariate models). These results imply that NCSE independently impaired achievement of favorable outcome in patients who may otherwise have had a reasonable prognosis. This assumption was further supported by the subgroup analysis. All seizures including NCSE were expectedly closely associated with larger amounts of blood within the initial CT scan (i.e., higher Fisher grade) [23, 24]. However, and possibly most surprisingly, both acute symptomatic NCSE as well as single self-limiting seizures were associated with unfavorable outcome exclusively in patients with a low Hunt-Hess grade, whereas these lost their predictive value in patients with a high Hunt-Hess grade.

Single self-limiting seizures and NCSE lead to a reduction of GCS: although single self-limiting seizures only lead to a transient reduction of GCS, NCSE leads to a continuous reduction of GCS either due to the seizing itself, or also due to its therapy (intravenous application of benzodiazepines, anesthetics, etc.). This renders the close evaluation of clinical signs of DCI impossible, and thus possibly the preventable secondary infarction due to DCI might be missed. This thesis might also explain why single seizures/NCSE primarily affected outcome in patients with low Hunt-Hess. These are the only patients who can be closely monitored for clinical signs of DCI, whereas patients with high Hunt-Hess grades commonly retain a low GCS independently of occurrence of single seizures or NCSE. However, whether NCSE poses a direct risk factor for unfavorable outcome, just impairs early diagnosis of DCI, or if they are just an expression of the severity of the disease itself cannot be determined in our study.

Between 2016 and 2020 there was an increase of EEGs performed as well as an increase of NCSE diagnosed emphasizing the importance of bearing-in-mind and evaluating presence of NCSE in case of unclear clinical deterioration to allow for earliest treatment possible. The vast majority of NCSE (irrespective of acute/remote origin) was first diagnosed between day 1 and 7 after aSAH in patients with unfavorable outcome (Fig. 2). The first patient (with unfavorable outcome) with his first NCSE at a later time-point was diagnosed at day 18 after aSAH. Diagnosis of epilepsy was found in similar rates as in previous studies [9, 13, 14]. Surprisingly, although only 16.3% of patients could be diagnosed with epilepsy, 25.5% of patients were still prescribed an ASM one year after onset of aSAH. Although prophylactic ASM has not conclusively been found to be associated with unfavorable outcome, there is evidence that phenytoin might impair outcome after aSAH [4, 11, 25]. Thus, clear documentation of the indication for the initiation of ASM as well as early/planned tapering of medication after the acute phase remain essential.

## Limitations

This study has several limitations. Because of the retrospective design, there was no standardized performance of EEGs. Whether an EEG was performed at all to allow for the diagnosis of NCSE was decided by the treating physician. Routine EEGs lasted 20 min. Longer EEGs or even continuous EEG monitoring would surely have increased the number of seizures/NCSE diagnosed. Particularly in poor grade aSAH, continuous EEG has shown to be beneficial their detection with up to 18% being diagnosed with nonconvulsive seizures and up to 13% being diagnosed with NCSE after a median monitoring duration of 3–4 days [17, 26, 27]. Other predictors of unfavorable outcome that can be extracted from continuous EEG and were unavailable in our study (i.e., absence of sleep architecture, total burden/duration of seizures/NCSE) [8, 17] might have improved the outcome prediction, but could also have led to earlier withdrawal of life-sustaining therapy. Some patients might have been treated with ASM even if a seizure was only suspected (i.e., due to an unclear motor event). This might have reduced the number of seizures/NCSE documented. However, in aSAH, NCSE has been found to be highly refractory to treatment, and the effect of ASM on the incidence of seizures in the early phase of aSAH remains controversial [4, 11, 19, 28, 29]. Furthermore, ASM might have even impaired outcome itself, as some ASM (especially phenytoin) have been shown to possibly pose a negative impact on outcome after aSAH [4, 11, 25]. On a long-term basis, as many patients were treated even after discharge, incidence of epilepsy is most probably underestimated.

Currently, there is no unified definition for the time limit of acute symptomatic seizures in aSAH. ILAE recommends acute symptomatic seizures to be defined as events occurring in close temporal relationship with an acute insult to the central nervous system [1]. Most commonly the time limit is set at 7 days after ictus. DCI occurs in around 30% of patients with aSAH [30, 31]. Particular in those cases in which DCI leads to infarction, the acute symptomatic time period most likely should be extended or even “restarted” to perfectly accommodate the recommendations proposed by the ILAE. However, although this might lower the false negative rate, it would unquestionably also increase the false positive rate of acute symptomatic seizure diagnosis. Lastly, unless a seizure or NCSE starts during an EEG its exact cortical origin cannot be determined in the absence of clearly lateralizing or localizing seizure manifestations, thus rendering the differentiation between acute symptomatic (i.e., due to DCI) and remote symptomatic seizure (i.e., due to the initial hemorrhage) vastly more difficult. In our cohort, the vast majority of NCSE occurred within the first 7 days; even more important, the vast majority of NCSE within the first 7 days occurred within the first 3 days after hemorrhage, thus before the start of the vasospasm phase (Fig. 2). These observations make a clear association of NCSE to DCI in comparison with the initial hemorrhage itself less likely. Altogether, in our cohort, the inclusion of later NCSE would most likely not have changed the results. Yet, we still believe that using a 7-day cut-off (although being somewhat arbitrary) will allow for better comparability with other studies and minimize the false positive rate.

## Conclusions

Seizures and NCSE remain a common occurrence in aSAH. Acute symptomatic NCSE in particular is independently associated with an unfavorable outcome. We propose that the ictal or postictal reduction of GCS might render close clinical evaluation for clinical signs of DCI impossible, and thus possibly leading to a delayed diagnosis of DCI and possibly preventable secondary infarction due to DCI. This thesis is supported by the subgroup analysis showing that seizures and NCSE primarily affected outcome in patients with a low Hunt-Hess grade while leaving patients with a high Hunt-Hess grade unaffected. Prospective studies with predefined protocols regarding the timing of EEG and CT imaging must be performed to validate this theory. Lastly, the performance of EEGs remains essential for outcome prediction in aSAH and treatment thereof, as NCSE incidence increased throughout the years with the increase of patients receiving an EEG.

## Data Availability

The data are available on reasonable request by the corresponding author.
